# ABCB1 and ABCG2 restricts the efficacy of gedatolisib (PF-05212384), a PI3K inhibitor in colorectal cancer cells

**DOI:** 10.1186/s12935-021-01800-7

**Published:** 2021-02-16

**Authors:** Changfu Liu, Wenge Xing, Haipeng Yu, Weihao Zhang, Tongguo Si

**Affiliations:** Department of Interventional Treatment, Tianjin Medical University Cancer Institute and Hospital, National Clinical Research Center for Cancer, Key Laboratory of Cancer Prevention and Therapy, Tianjin, Tianjin’s Clinical Research Center for Cancer, Tianjin, 300060 China

**Keywords:** Gedatolisib, Colorectal cancer (CRC), ABCB1, ABCG2, Drug resistance, Substrates

## Abstract

**Background:**

Overexpression of ABC transporters is a big challenge on cancer therapy which will lead cancer cells resistance to a series of anticancer drugs. Gedatolisib is a dual PI3K and mTOR inhibitor which is under clinical evaluation for multiple types of malignancies, including colorectal cancer. The growth inhibitory effects of gedatolisib on colorectal cancer cells have been specifically studied. However, the role of ABC transporters on gedatolisib resistance remained unclear. In present study, we illustrated the role of ABC transporters on gedatolisib resistance in colorectal cancer cells.

**Methods:**

Cell viability investigations of gedatolisib on colorectal cancer cells were determined by MTT assays. The verapamil and Ko143 reversal studies were determined by MTT assays as well. ABCB1 and/or ABCG2 siRNA interference assays were conducted to verify the role of ABCB1- and ABCG2-overexpression on gedatolisib resistance. The accumulation assays of gedatolisib were conducted using tritium-labeled paclitaxel and mitoxantrone. The effects of gedatolisib on ATPase activity of ABCB1 or ABCG2 were conducted using PREDEASY ATPase Kits. The expression level of ABCB1 and ABCG2 after gedatolisib treatment were conducted by Western blotting and immunofluorescence assays. The well-docked position of gedatolisib with crystal structure of ABCB1 and ABCG2 were simulated by Autodock vina software. One-way ANOVA was used for the statistics analysis.

**Results:**

Gedatolisib competitively increased the accumulation of tritium-labeled substrate-drugs in both ABCB1- and ABCG2-overexpression colorectal cancer cells. Moreover, gedatolisib significantly increased the protein expression level of ABCB1 and ABCG2 in colorectal cancer cells. In addition, gedatolisib remarkably simulated the ATPase activity of both ABCB1 and ABCG2, suggesting that gedatolisib is a substrate drug of both ABCB1 and ABCG2 transporters. Furthermore, a gedatolisib-resistance colorectal cancer cell line, SW620/GEDA, was selected by increasingly treatment with gedatolisib to SW620 cells. The SW620/GEDA cell line was proved to resistant to gedatolisib and a series of chemotherapeutic drugs, except cisplatin. The ABCB1 and ABCG2 were observed overexpression in SW620/GEDA cell line.

**Conclusions:**

These findings suggest that overexpression of ABCB1 and ABCG2 may restrict the efficacy of gedatolisib in colorectal cancer cells, while co-administration with ABC transporter inhibitors may improve the potency of gedatolisib.

## Background

Colorectal cancer (CRC) is one of the most commonly diagnosed cancers all over the world, which ranked at the third place in both men and women [1, 2]. There are more than one million newly diagnosed patients in which 0.6 million deaths per year [3]. The overall survival of CRC is highly dependent on the diagnosed stage. The prognosis of diverse stage of CRC is in different, patients diagnosed with stage I CRC always obtain a good prognosis with 90% 5-year overall survival (OS), however, the 5-year OS of stage IV CRC is only 10%. Though surgery has been treated as a necessary approach in early stage CRC, consolidation chemotherapy is highly recommended to improve the tumor microenvironment (TME) and to eliminate minimal residual disease (MRD). However, drug-resistance remains one of the deadlocks for the low survival rates of CRC patients [4].

The phosphoinositide 3-kinases (PI3Ks) plays a central role in cancer proliferation and survival [5–7]. PI3Ks are lipid kinases which could phosphorylate phosphatidylinositol 4,5-bisphosphate (PIP2) by 3-OH position of inositol ring to produce phosphatidylinositol 3,4,5-trisphosphate (PIP3), subsequently activates downstream pathways, such as PI3K/Akt/mTOR (PAM) pathway, in multiple types of cancer, including CRC [8, 9]. Singularly activation of PAM pathways, for example, activation of Akt, could contribute to cell cycle progression through regulating glycogen synthesis kinase 3β (GSK-3β), as well as cyclin D1, which would maintain cell survival by antagonizing Bcl-2-antagonist of cell death (BAD), and upregulate survival mTOR phosphorylation [9, 10]. Hence, PI3Ks-related proteins present as attractive treatment targets for malignancy.

The U.S. Food and Drug Administration (FDA) has approved idelalisib, a PI3K inhibitor, for management relapsed follicular B-cell non-Hodgkin lymphoma, relapsed small lymphocytic lymphoma, or chronic lymphocytic leukemia [11]. Besides, Piqray (alpelisib) from Novartis has been approved by FDA for the co-treatment with fulvestrant for PIK3CA-mutant HR-positive/HER2-negative late-stage/metastasis breast cancer, and this is the first PI3K inhibitor approved for breast cancer treatment [12]. In addition, a series of clinical and preclinical progresses are ongoing to evaluate the efficacy of different PI3K inhibitors in multiple types of cancer [13, 14]. Gedatolisib (PF-05212384, PKI-587), is a potent dual PI3Kα, PI3Kγ, and mTOR inhibitor, which is under clinical evaluation [15, 16]. Though gedatolisib is effective and safe in administrating for specific cancer types, its resistance mechanisms remained unclear.

Multidrug resistance (MDR) is a phenomenon which cancer cells are resistant to structural- and target-unrelated anticancer drugs, which finally lead to the failure of cancer therapy [17, 18]. A few mechanisms are involved in MDR in cancer, including reducing in apoptosis, the repairing of DNA damage, or the alteration of anti-cancer drug metabolism [19]. ATP-binding cassette (ABC) transporters, contain seven subfamilies from ABCA to ABCG, are one of the largest protein families, which regulate physiological substrates to maintain physiological and pharmacological functions [20]. More importantly, ABC transporters also play a key role in MDR. Overexpression of specific ABC transporters will restrict the efficacy of certain anti-cancer drugs, by “pumping” these drugs out of cancer cells. In present study, we evaluated the role of ABCB1 and ABCG2 in process of gedatolisib resistance in colorectal cancer cells.

## Methods

### Chemicals

Gedatolisib (purity: 99.09%) was purchased from Selleck Chemicals (Houston, TX, USA). Paclitaxel (purity: 99.02%), doxorubicin (purity: 99.37%), vincristine (purity: 99.19%), mitoxantrone hydrochloride (purity: 99.9%), topotecan hydrochloride (purity: 100.0%), and cisplatin (purity: 99.62%) were purchased from Energy Chemicals (Shanghai, China). Verapamil hydrochloride (purity: 99.95%) and Ko143 (purity: 99.79%) were purchased from MedChemExpress (NJ, USA). Tritium-labeled paclitaxel and mitoxantrone were obtained from SuYa Medicine (Beijing, China). Fetal bovine serum (FBS), Dulbecco’s modified Eagle’s Medium (DMEM), penicillin/streptomycin, and trypsin were purchased from Corning Incorporated (Corning, NY, USA). G418, DMSO, 3-[4,5-dimethylthiazol-yl]-2,5-diphenyltetrazolium bromide (MTT), propidium iodide (PI), and paraformaldehyde, were purchased from Beyotime Biotechnology (Shanghai, China). Antibody of GAPDH, ABCB1, and ABCG2, Alexa Fluor 488 conjugated goat anti-mouse IgG secondary antibody, HRP-conjugated goat anti-mouse IgG secondary antibody were purchased from Cell Signaling Technology (Danvers, MA, USA). All other chemicals were obtained from Sigma Chemical Co (St. Louis, MO, USA).

### Cell lines and cell culture

The human colorectal cancer cell line SW620 and LS180 were purchased from Cell Resource Center, Peking Union Medical College (Beijing, China). The ABCB1-overexpresison SW620/ADR cell line was selected as previously described [21]. Briefly, SW620 cells were grown in complete DMEM medium, stepwise exposure to doxorubicin, the beginning exposure concentration was 30 nM, which finally increased to 300 nM for 3 months. Finally, a clonal cell line that grew in the presence of 0.3 μM doxorubicin were isolated as SW620/ADR. The ABCG2-overexpression LS180/MX cell line was selected by increasingly exposure to mitoxantrone from 1 μM to 80 μM as previously described [22]. Finally, a clonal cell line that grew in the presence of 80 μM mitoxantrone was isolated as LS180/MX. HEK293/pcDNA3.1, HEK293/ABCB1, and HEK293/ABCG2 cells were transfected with pcDNA3.1 vector or vector with full length of ABCB1 or ABCG2, which were kindly obtained from Zhejiang University (Zhejiang, China). HEK293/ABCB1, and HEK293/ABCG2 cells were selected with complete medium containing of G418 (2 mg/ml). All of the cell lines were cultured in 10% FBS DMEM medium containing 5% CO_2_ and humid atmosphere.

### Cell viability analysis and reversal study by MTT assay

MTT assays were conducted to evaluate gedatolisib on parental, ABCB1-, and ABCG2-overexpression cells proliferation. In brief, 4000 cells were seeded into 96 well plates in 160 μL of total medium for overnight, then treated with different concentrations of gedatolisib for another 72 h at 37 ℃ containing 5% CO_2_ humid circumstance. For verapamil and Ko143 reversal study, verapamil (5 μM) or Ko143 (5 μM) was added 2 h priority to gedatolisib administration. MTT (5 mg/ml) was added for another 4 h further incubation, and DMSO was used to dissolve the formazan. A Bio-Rad microplate reader iMark (Hercules, CA, USA) was used to read the OD value and a Graphpad software was used to draw the concentration-proliferation curves.

### Tritium-labeled paclitaxel and mitoxantrone accumulation assay

[^3^H]-Paclitaxel and [^3^H]-mitoxantrone were used as probes in this study. Briefly, 1,000,000 cells were seeded into 6-well plates for overnight. Different concentrations of Gedatolisib (3 μM and 6 μM) were added for 2 h before adding into [^3^H]-paclitaxel in SW620 and SW620/ADR cells, or [^3^H]-mitoxantrone in LS180 and LS180/MX cells. After administrating with probes, cells were harvested and wash with iced PBS twice. Subsequently, cells were transferred into scintillation vials containing 5 mL scintillation fluid. A MicroBeta2 (PerkinElmer, NY, USA) was used to measure the radioactivity.

### ATPase activity of ABCB1 and ABCG2

The influence of gedatolisib on ABCB1- and ABCG2-medated ATP hydrolysis were evaluated by PREDEASY ATPase Kits (TEBU-BIO nv, Boechout, Belgium) with modified protocols. Briefly, ABCB1 or ABCG2 membranes were thawed and diluted carefully at 4 ℃. Na_3_VO_4_ was used to inhibit ATPase activity. Various concentrations of gedatolisib were incubated with membranes for 5 min. Mg^2+^ ATP (5 mM) was used to trigger the reaction. Luminescence signals of P_i_ were initiated and measured after incubation at 37 ℃ for 40 min with brief mixing. The changes of relative light units were determined by comparing Na_3_VO_4_-treated samples with gedatolisib treated groups.

### Western blot and immunofluorescence assays

Western blot assays were used to evaluate the alteration at expression level of ABCB1 and ABCG2 changed by gedatolisib in colorectal cancer cell lines. Briefly, cells were treated with or without gedatolisb for 24, 48, and 72 h. RIPA cell lysis buffer was used to lyse cells and extract total protein. After standardization of protein concentration, polyacrylamide gel electrophoresis (SDS-PAGE) was used to loading and separation the proteins, which were subsequently transferred into PVDF membrane. Non-fat milk (5 mg/ml) was used to blocking the non-specific proteins, and primary antibodies (ABCB1 or ABCG2) were incubated for overnight in 4 ℃. The second antibody (HRP-conjugated) was incubated for 1 h and ECL was used to visualize the protein bands. GAPDH was used as a loading control. An Image J software was used to analysis the relative protein expression level.

Immunofluorescence assays were used to detected the influence of gedatolisib on ABCB1 or ABCG2 subcellular localization. Briefly, cells were seeded into 6-well plates for overnight, and treated with gedatolisib (6 μM) for 72 h. Then, cells were washed twice by iced-cold PBS and fixed by 4% methanol. BSA (5 mg/ml) were used to blocking the non-specific proteins and primary antibodies (ABCB1 or ABCG2) were incubated for 2 h as it is recommended in a commercial protocol. Second antibodies were incubated for another 1 h. PI was used to counter the nuclei. A Leica DMi8 fluorescence microscope (Leica, Wetzlar, Germany) was used to collect the images.

### Establishment of gedatolisib-resistance colorectal cancer cells, SW620/GEDA

Gedatolisib was increasingly added into SW620 cells for 3 months. SW620/GEDA was established. Most cells survived after treated with gedatolisib (5 μM). MTT assays were used to evaluate the resistance spectrum as abovementioned. The expression level of ABCB1 and ABCG2 of SW620/GEDA were evaluated by Western blot.

### ABCB1 and/or ABCG2 siRNA interference assays

SW620/GEDA cell line (5 × 10^5^) were plated in 6-well plates for overnight. Subsequently, cells were incubated with the medium of Opti-MEM with Lipo3000 reagent, ABCB1 siRNA (50 nM), and/or ABCG2 siRNA (50 nM), or scrambled control siRNA for 6 h in cellular incubator. The cells were further incubated for another 72 h. Western blot assays were used to verify the expression level of ABCB1 and/or ABCG2. To determine the sensitivity of gedatolisib, doxorubicin, paclitaxel, mitoxantrone, and topotecan associated with ABCB1 and/or ABCG2, the siRNA-transfected cells were exposed to those drugs for 72 h, with subsequently detection by MTT assay.

### Molecule docking of gedatolisib with modeled structure ofABCB1 and ABCG2

The crystal structure of ABCB1 (PDB: 6FN1), and ABCG2 (PDB: 6ETI) was used for docking with gedatolisib by Autodock vina software. The receptor grid was centered at center_x = 148.147, center_y = 130.984, center_z = 130.234, size_x = 25, size_y = 25, size_z = 25 for ABCB1, and center_x = 134.122, center_y = 131.764, center_z = 135.233, size_x = 25, size_y = 25, size_z = 25. The exhaustiveness was set at 50 for both proteins.

### Statistical analysis

All data are shown as the mean ± SD and analyzed using one-way ANOVA. All experiments were repeated at least three independent experiments. Differences were considered significant when P vale less than 0.05.

## Results

### Gedatolisib is less effective in inhibiting the proliferation of SW620/ADR or LS180/MX cells, which overexpression ABCB1 or ABCG2

**Fig. 1:**
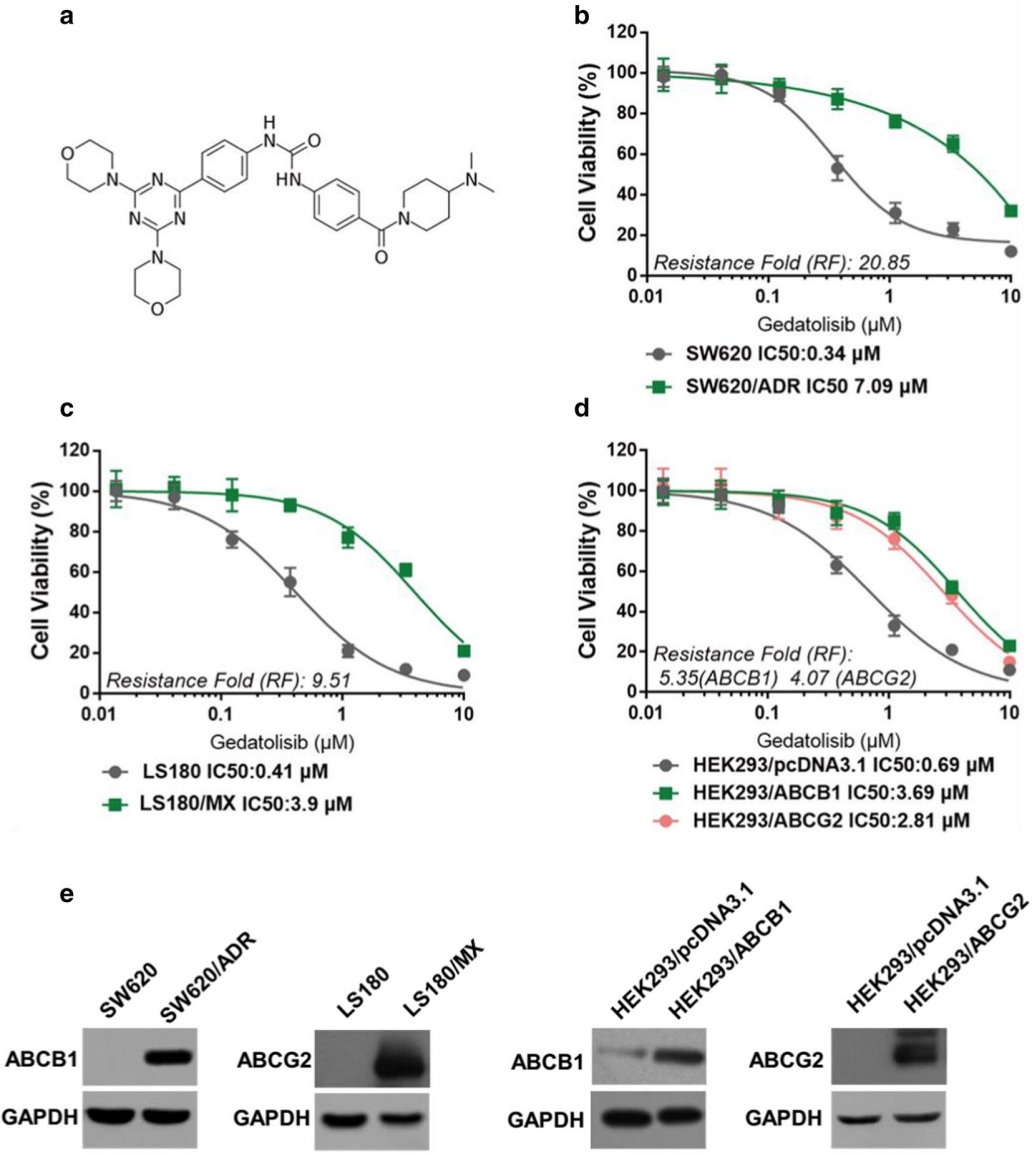
Anti-proliferation effects of gedatolisib in parental and ABCB1- or ABCG2-overexpression cells. **a** Chemical structure of gedatolisib. **b** Dose-viability curves of SW620 and SW620/ADR cells after treatment with different concentration of gedatolisib for 72 h. **c** Dose-viability curves of LS180 and LS180/MX cells after treatment with different concentration of gedatolisib for 72 h. **d** Dose-viability curves of HEK293/pcDNA3.1, HEK293/ABCB1, and HEK293/ABCG2 cells after treatment with different concentration of gedatolisib for 72 h. **e** Confirmation of overexpression of ABCB1 or ABCG2 in drug-selected and DNA-transfected cell lines. Data were shown with mean ± SD, which is representative for at least three independent experiments

Firstly, we conducted MTT assays to evaluate the cytotoxic effect of gedatolisib in different colorectal cancer cells. Figure 1 showed the significant inhibitory effects of gedatolisib on sensitive SW620 or LS180 cells after 72 h incubation. However, drug-resistant SW620/ADR or LS180/MX cells were resistance to gedatolisib (The SW620/ADR cells were derived from SW620 cells by increasingly treatment with doxorubicin. The LS180/MX cells were derived from LS180 cells by increasingly treatment with mitoxantrone). The IC_50_ value of gedatolisib in parental SW620 cells was 0.34 μM, while in resistance SW620/ADR cells, the IC_50_ value of gedatolisib was 7.09 μM, which is 20.85 folds than that in SW620 cells (Fig. 1b). As illustrated in Fig. 1c, the IC_50_ value of gedatolisib in parental LS180 cells was 0.41 μM, and that in resistance LS180/MX cells was 3.9 μM, the resistance folds (RF) were 9.51. The resistance fold was calculated via dividing the IC_50_ values of resistance cells (SW620/ADR or LS180/MX) by the IC_50_ values of sensitive cells (SW620 or LS180). These results indicated that the overexpression of ABCB1 or ABCG2 may restrict the anticancer effect of gedatolisib.

### Gedatolisib is less effective on inhibiting the proliferation of HEK293/ABCB1 or HEK293/ABCG2 cells

To further verify that ABCB1 and ABCG2 could confer resistance to gedatolisib, we evaluated the antiproliferation effects of gedatolisib in ABCB1- and ABCG2-gene-transfected cells (HEK293/ABCB1 and HEK293/ABCG2). As it is shown in Fig. 1d, the IC_50_ value of gedatolisib in HEK293/ABCB1 cells (3.69 μM) was significantly higher than that in parental HEK293/pcDNA3.1 cells (0.69 μM). Moreover, the IC_50_ value of gedatolisib in HEK293/ABCG2 cells (2.81 μM) was significantly higher than that in parental HEK293/pcDNA3.1 cells. These results further confirmed that overexpression of ABCB1 or ABCG2 was related with gedatolisib resistance in colorectal cancer cells.

### Documented ABC transporter inhibitors significantly reverse gedatolisib resistance in ABC transporter-overexpression cells

**Fig. 2:**
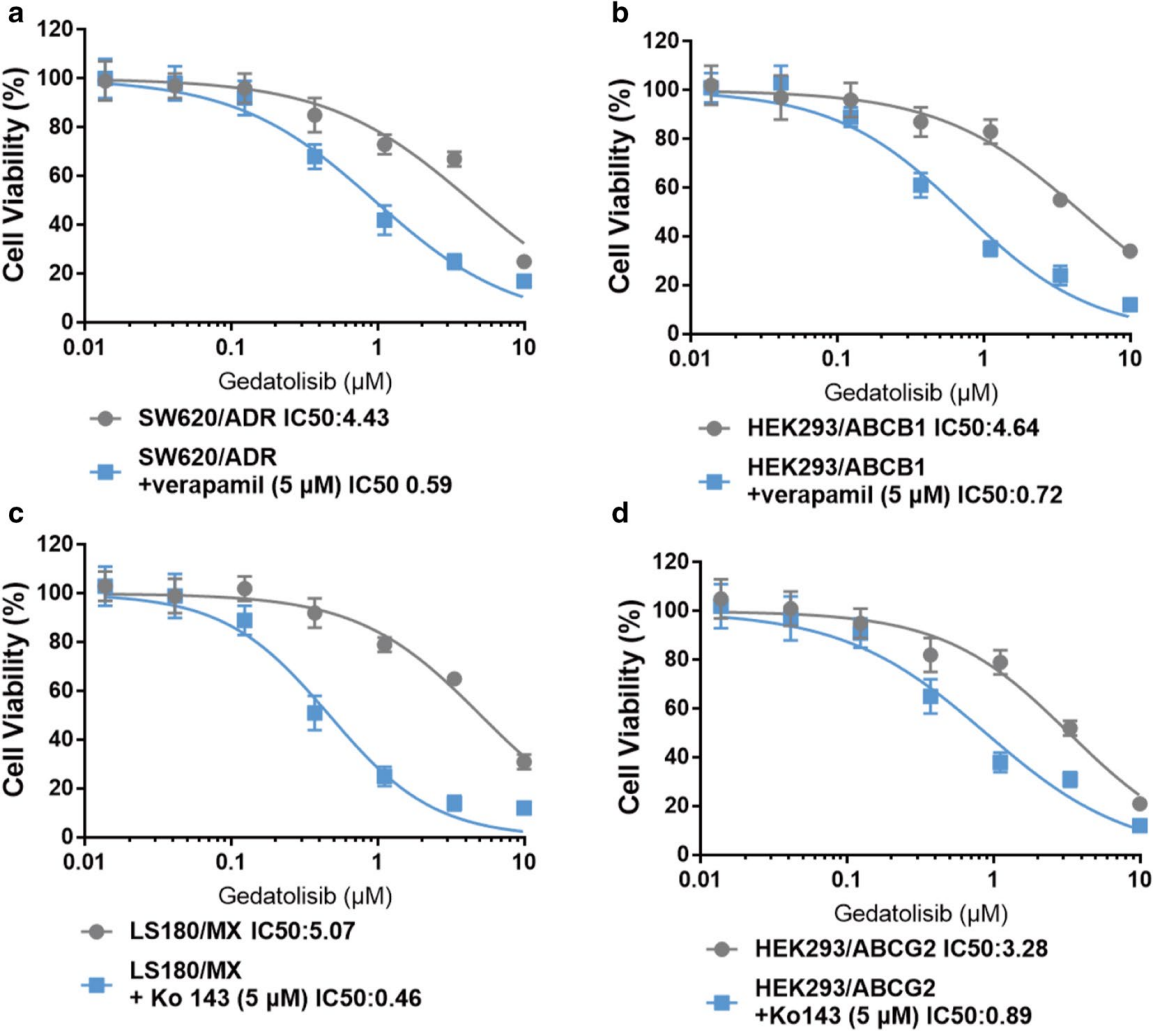
ABCB1 and ABCG2 reversal agents significantly reverse gedatolisib resistance. **a** Dose-viability curves of SW620/ADR cells after treatment with different concentration of gedatolisib only or gedatolisib plus verapamil (5 μM) for 72 h. **b** Dose-viability curves of HEK293/ABCB1 cells after treatment with different concentration of gedatolisib only or gedatolisib plus verapamil (5 μM) for 72 h. **c** Dose-viability curves of LS180/MX cells after treatment with different concentration of gedatolisib only or gedatolisib plus Ko143 (5 μM) for 72 h. **d** Dose-viability curves of HEK291/ABCG2 cells after treatment with different concentration of gedatolisib only or gedatolisib plus Ko143 (5 μM) for 72 h. Data were shown with mean ± SD, which is representative for at least three independent experiments

Verapamil and Ko 143 are reported as ABCB1 or ABCG2 potent inhibitor [23, 24]. To evaluate whether these inhibitors could reverse gedatolisib resistance in ABCB1- or ABCG2- overexpression cells, we conducted reversal studies using aforementioned inhibitors with reported concentrations. As it is shown in Fig. 2a, b, after treatment with verapamil (5 μM), a specific ABCB1 inhibitor, the IC_50_ value of gedatolisib in SW620/ADR cells and HEK293/ABCB1 cells decreased significantly compared with control group. In addition, Ko143 (5 μM) significantly reverse gedatolisib resistance in LS180/MX cells and HEK293/ABCG2 cells (Fig. 2c, d).

### Gedatolisib increased the accumulation of ABCB1 and ABCG2 substrate drugs

To get thorough comprehension into the mechanism of action on above phenomenon, the tritium-labeled substates accumulation assays were conducted.

**Fig. 3:**
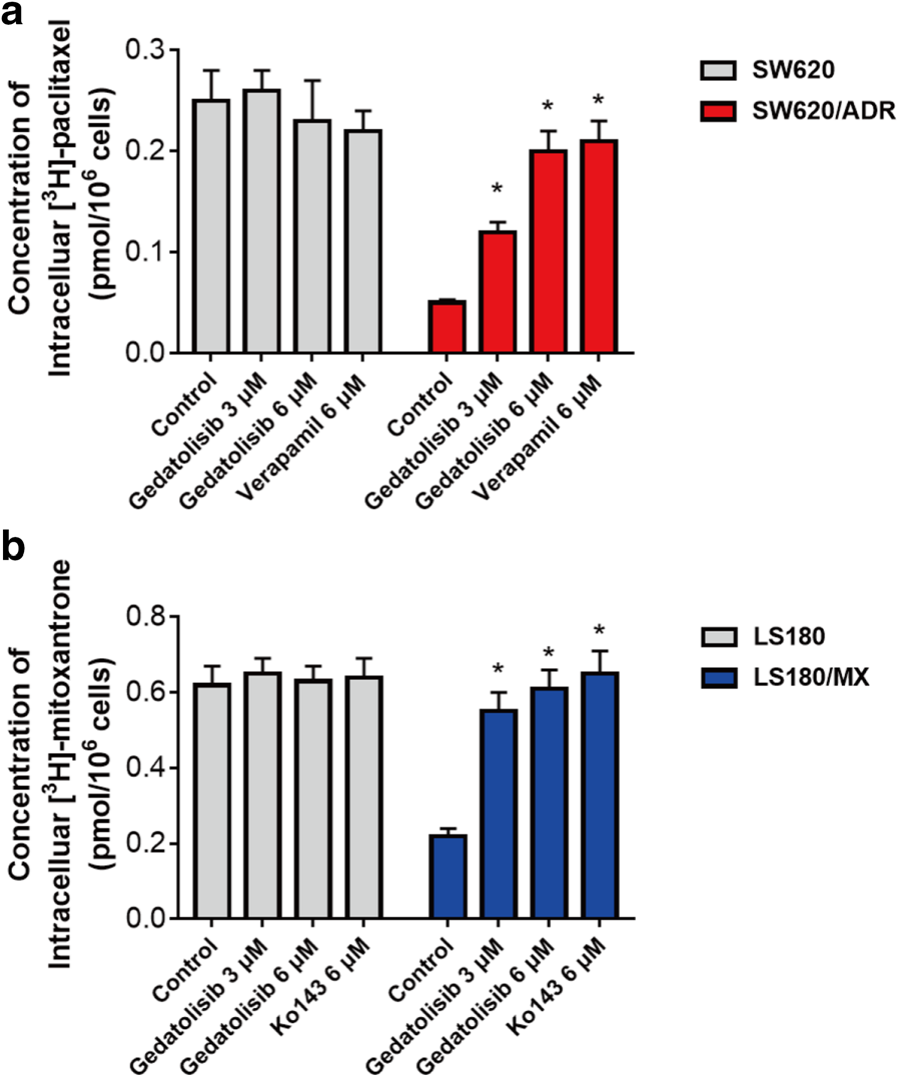
Gedatolisib significantly increased the tritium-labeled substrate-drugs accumulation in ABCB1- and ABCG2-overexpression colorectal cancer cells. **a** The accumulation of [^3^H]-paclitaxel in parental SW620 cells or resistance SW620/ADR cells after treatment with gedatolisib for 2 h. **b** The accumulation of [^3^H]-mitoxantrone in parental LS180 cells or resistance LS180/MX cells after treatment with gedatolisib for 2 h. Data were shown as mean ± SD, which is representative for three independent experiments. Asterisk presents when p value is < 0.05

As it is documented that paclitaxel or mitoxantrone is a substrate of ABCB1 or ABCG2 respectively, we therefore used [^3^H]-paclitaxel and [^3^H]-mitoxantrone to evaluated the efflux function of ABCB1 or ABCG2 on gedatolisib accumulation in SW620/ADR and LS180/MX cells. As it is shown in Fig. 3a, the accumulation level of [^3^H]-paclitaxel was significantly increased after short-time (2 h) co-incubation with gedatolisib (3 and 6 μM) in SW620/ADR cells. However, in parental SW620 cells, there was no significantly alteration of [^3^H]-paclitaxel accumulation level between gedatolisib treatment groups and control group. Moreover, the intracellular concentrations of [^3^H]-mitoxantrone were also up-regulated significantly after treatment with gedatolisib (3 and 6 μM), while in parental LS180 cells, similar positive results were not found (Fig. 3b). These results indicated that gedatolisib is a potential substrate of ABCB1 and ABCG2, which could compete with substrate of the ABC transporters, resulted in increased accumulation of tritium-labeled substrate drugs ([^3^H]-paclitaxel or [^3^H]-mitoxantrone).

### Gedatolisib stimulated the ATPase of ABCB1 and ABCG2

**Fig. 4:**
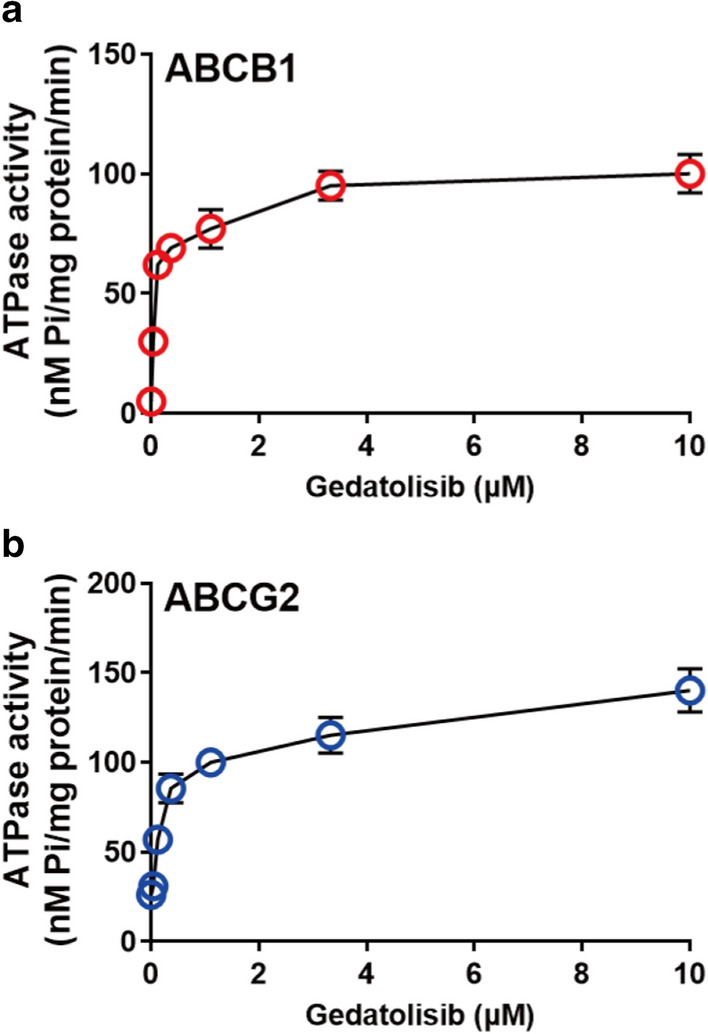
Gedatolisib stimulated the ATPase of ABCB1 and ABCG2. **a** Effect of gedatolisib on the ATPase activity of ABCB1. **b** Effect of gedatolisib on the ATPase activity of ABCG2. Data were shown as mean ± SD, which is representative for three independent experiments. Asterisk presents when p value is < 0.05

ABCB1- and ABCG2-mediated ATP hydrolysis were determined by a commercial reagent kit. As it is shown in Fig. 4a, b, gedatolisib significantly stimulated the ATPase of both ABCB1 and ABCG2 in dose-dependent manners. This result further indicated that gedatolisib could be a substrate of ABCB1 or ABCG2.

### Gedatolisib increased the expression level of ABCB1 or ABCG2 in drug resistance CRC cell lines without altering the subcellular localization of ABCB1 or ABCG2

**Fig. 5:**
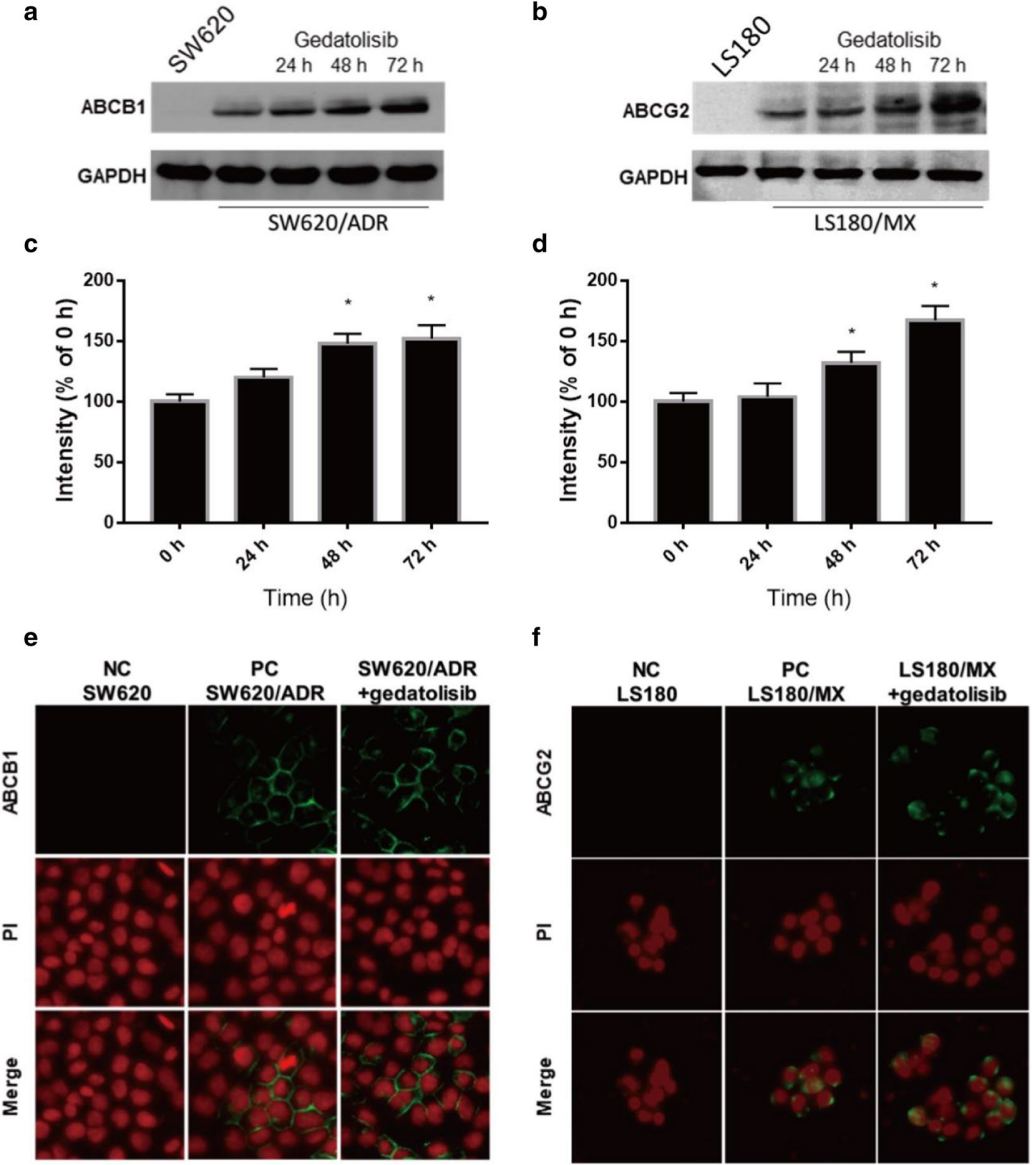
Gedatolisib increased the expression level of ABCB1 or ABCG2 in drug resistance cells without altering the subcellular localization of ABCB1 or ABCG2. **a** Protein expression level of ABCB1 in SW620/ADR cells after treatment with gedatolisib (1 μM) for 24, 48, and 72 h. **b** Protein expression level of ABCG2 in LS180/MX cells after treatment with gedatolisib (1 μM) for 24, 48, and 72 h. Relative expression level of ABCB1 (**c**) and ABCG2 (**d**) after treatment with 1 μM of gedatolisib. **e** Subcellular localization of ABCB1 in SW620/ADR cells after treatment with gedatolisib (1 μM) for 72 h. **f** Subcellular localization of ABCG2 in LS180/MX cells after treatment with gedatolisib (1 μM) for 72 h. Green represents ABCB1 or ABCG2, red shows the nuclei which is dyed by PI. Scale bar 50 μm. Data were shown as mean ± SD, which is representative for three independent experiments. Asterisk presents when p value is < 0.05

Since gedatolisib had limit effect to inhibit ABCB1- and ABCG2-overexpression cancer cells, the potential mechanisms could be related with the upregulation of ABC transporter expression level. As it is shown in Fig. 5a, the ABCB1 expression level in SW620/ADR cells were significantly increased after treatment with 1 μM of gedatolisib for 72 h. Moreover, in LS180/MX cells, the ABCG2 expression level was also significantly up-regulated after treatment 1 μM of gedatolisib for 72 h (Fig. 5b). These results indicated that overexpression of ABCB1 or ABCG2 is a major element which resulted in acquired gedatolisib resistance. In addition, as our immunofluorescence results illustrated, gedatolisib did not significantly altered the subcellular localization of ABCB1 or ABCG2 in drug-resistant colorectal cancer cells (Fig. 5c, d).

### Gedatolisib could partially reverse ABCB1 or ABCG2-mediated MDR

It is documented that a series of reversal agents are substrates of ABC transporters, especially ABCB1 and ABCG2. To determine whether low toxic concentrations of gedatolisib could reverse ABCB1- or ABCG2-mediated MDR in colorectal cancer cells, reversal studies were conducted by MTT assays. As it is shown in Tables 1 and 2, low cytotoxic concentrations gedatolisib could partially reverse ABCB1- or ABCG2-mediated MDR in colorectal cancer cells. Specifically, gedatolisib (0.1 and 0.2 μM) significantly decrease the IC_50_ values of paclitaxel, doxorubicin, and vincristine in SW620/ADR cells, while in SW620 cells, gedatolisib could not influence the IC_50_ values of paclitaxel, doxorubicin, or vincristine. In addition, gedatolisib significantly down-regulated the IC_50_ values of mitoxantrone, topotecan, and SN-38 in LS180/MX cells, while in parental LS180 cells, gedatolisib could not impact on corresponding IC_50_ values of mitoxantrone, topotecan, or SN-38. Moreover, gedatolisib could not alter the IC_50_ values of cisplatin, which was used as a negative control in this assay. These results indicated that gedatolisib could partially reverse ABCB1- and ABCG2-mediated MDR in colorectal cancer cells.

**Table 1:** Gedatolisib partially reversed ABCB1-medaited MDR

Drug	IC_50_ ± SD(RF)	
	SW620 (μM)	SW620/ADR (μΜ)
*Doxorubicin*	0.042 ± 0.009 (1.00)	10.653 ± 0.600 (253.64)
+ Gedatolisib (0.1 μM)	0.043 ± 0.009 (1.024)	9.767 ± 0.424 (232.55)
+ Gedatolisib (0.2 μM)	0.039 ± 0.010 (0.928)	6.431 ± 0.041 (153.12)*
+ Verapamil (1 μM)	0.038 ± 0.007 (0.905)	0.412 ± 0.030 (9.80)*
*Paclitaxel*	0.038 ± 0.005 (1.00)	5.422 ± 0.556 (142.68)
+ Gedatolisib (0.1 μM)	0.040 ± 0.004 (1.05)	4.379 ± 0.234 (115.24)*
+ Gedatolisib (0.2 μM)	0.037 ± 0.006 (0.97)	3.273 ± 0.121 (86.13)*
+ Verapamil (1 μM)	0.042 ± 0.004 (1.10)	0.090 ± 0.082 (2.37)*
*Vincristine*	0.098 ± 0.009 (1.00)	12.472 ± 1.263 (127.27)
+ Gedatolisib (0.1 μM)	0.088 ± 0.008 (0.90)	10.863 ± 1.463 (110.85)*
+ Gedatolisib (0.2 μM)	0.091 ± 0.009 (0.93)	5.324 ± 0.561 (54.33)*
+ Verapamil (1 μM)	0.087 ± 0.009 (0.89)	0.231 ± 0.042 (2.36)*
*Cisplatin*	3.234 ± 0.194 (1.00)	3.536 ± 0.211 (1.09)
+ Gedatolisib (0.1 μM)	3.354 ± 0.213 (1.04)	3.743 ± 0.263 (1.13)
+ Gedatolisib (0.2 μM)	3.453 ± 0.253 (1.07)	3.243 ± 0.373 (0.8)
+ Verapamil (1 μM)	3.244 ± 0.210 (1.00)	3.543 ± 0.262 (1.07)

Resistance fold (RF) was calculated from dividing the IC_50_ values of SW620/ADR cells by the IC_50_ values of SW620 cells in the presence or absence of gedatolisib or verapamil

*Presents when p value is < 0.05. Data was showed with mean ± SD, which is representative for at least three independent experiments

**Table 2:** Gedatolisib partially reversed ABCG2-medaited MDR

Drug	IC_50_ ± SD^1^(RF^2^)	
	LS180 (μM)	LS180/MX (μΜ)
*Mitoxantrone*	0.112 ± 0.007 (1.00)	19.235 ± 2.639 (171.74)
+ Gedatolisib (0.1 μM)	0.132 ± 0.007 (1.18)	17.735 ± 2.489 (158.35)
+ Gedatolisib (0.2 μM)	0.109 ± 0.012 (0.97)	14.824 ± 1.112 (132.36)*
+ Ko143 (1 μM)	0.121 ± 0.009 (1.08)	0.616 ± 0.058 (5.5)*
*Topotecan*	0.098 ± 0.007 (1.00)	25.683 ± 2.031 (262.07)
+ Gedatolisib (0.1 μM)	0.089 ± 0.009 (0.90)	24.583 ± 1.543 (250.85)*
+ Gedatolisib (0.2 μM)	0.087 ± 0.008 (0.89)	18.632 ± 2.332 (190.12)*
+ Ko143 (1 μM)	0.091 ± 0.009 (1.02)	0.319 ± 0.054 (3.58)*
*SN-38*	0.111 ± 0.010 (1.00)	8.299 ± 0.923 (74.76)
+ Gedatolisib (0.1 μM)	0.107 ± 0.012 (0.96)	6.990 ± 0.777 (62.97)*
+ Gedatolisib (0.2 μM)	0.106 ± 0.011 (0.95)	5.676 ± 0.859 (51.62)*
+ Ko143 (1 μM)	0.112 ± 0.011 (1.01)	0.277 ± 0.031 (2.50)*
*Cisplatin*	2.511 ± 0.356 (1.00)	2.445 ± 0.390 (0.97)
+ Gedatolisib (0.1 μM)	2.620 ± 0.321 (1.04)	2.214 ± 0.342 (0.88)
+ Gedatolisib (0.2 μM)	2.582 ± 0.323 (1.03)	2.564 ± 0.367 (1.02)
+ Ko143 (1 μM)	2.391 ± 0.398 (0.95)	2.523 ± 0.369 (1.01)

Resistance fold (RF) was calculated from dividing the IC_50_ values of LS180/MX cells by the IC_50_ values of LS180 cells in the presence or absence of gedatolisib or Ko143

*Presents when p value is < 0.05. Data was showed with mean ± SD, which is representative for at least three independent experiments

### Overexpression of ABCB1 and ABCG2 may contribute to the resistance mechanisms of gedatolisib-resistance cell line SW620/GEDA

**Fig. 6:**
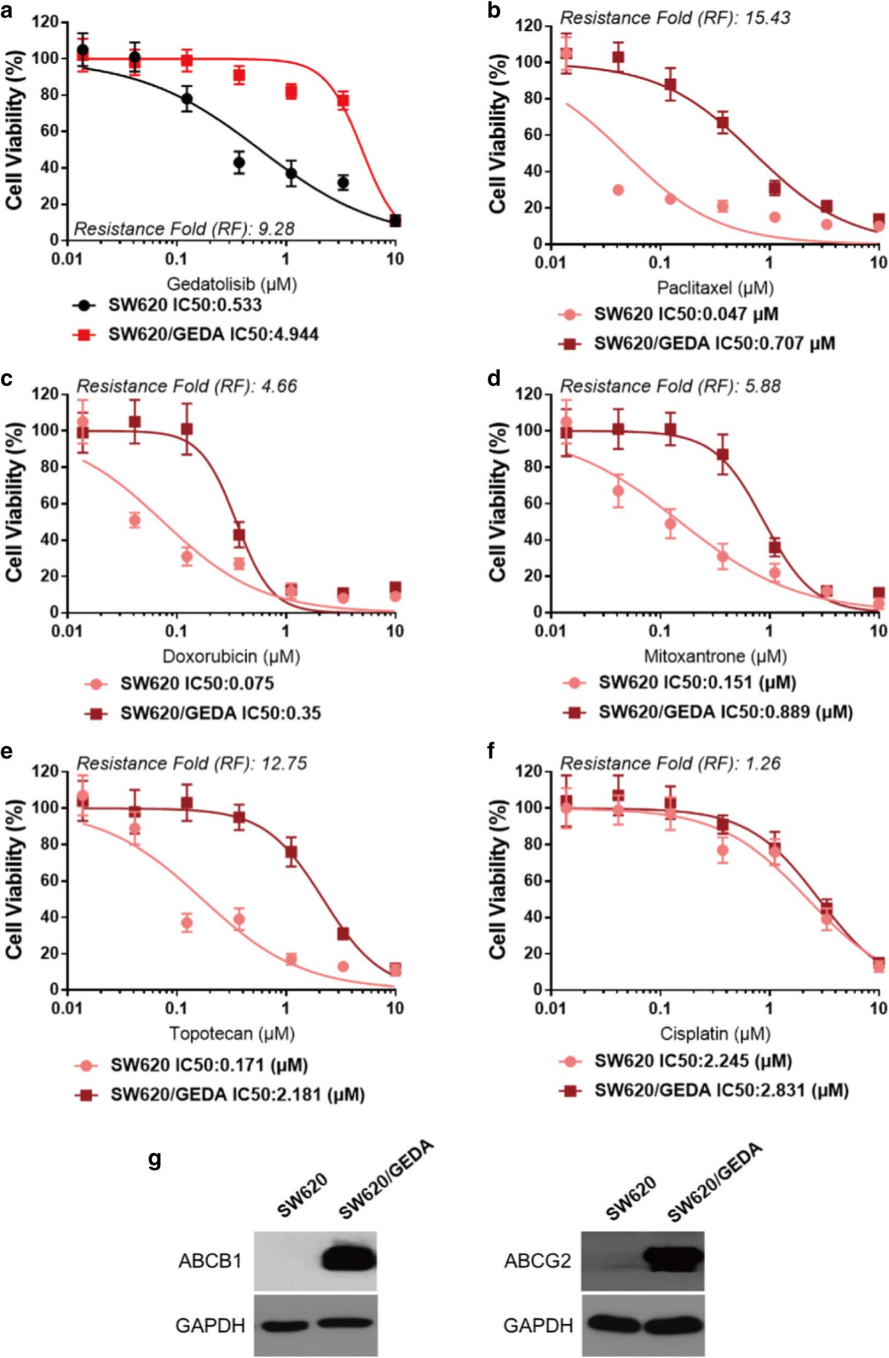
Evaluation of SW620/GEDA, a resistance cell line created by gedatolisib. **a** Dose-viability curves of SW620 and SW620/GEDA cells after treatment with different concentration of gedatolisib for 72 h. **b** Dose-viability curves of SW620 and SW620/GEDA cells after treatment with different concentration of paclitaxel for 72 h. **c** Dose-viability curves of SW620 and SW620/GEDA cells after treatment with different concentration of doxorubicin for 72 h. **d** Dose-viability curves of SW620 and SW620/GEDA cells after treatment with different concentration of mitoxantrone for 72 h. **e** Dose-viability curves of SW620 and SW620/GEDA cells after treatment with different concentration of topotecan for 72 h. **f** Dose-viability curves of SW620 and SW620/GEDA cells after treatment with different concentration of cisplatin for 72 h. **g** The expression of ABCB1 and ABCG2 in SW620/GEDA cell line. Data were shown as mean ± SD, which is representative for three independent experiments. Asterisk presents when p value is < 0.05

To get more insight into the mechanisms of action on gedatolisib resistance in colorectal cancer cells, we created gedatolisib-resistance colorectal cancer cells by adding increasing concentrations of gedatolisib into SW620 cells. Finally, SW620/GEDA was successfully established after 3 months. As it is shown in Fig. 6a, the IC_50_ value of gedatolisib in SW620/GEDA cells was 3 μM, which is 10 folds higher than that in parental SW620 cells. Next, we conducted MTT assays to evaluate the response of SW620/GEDA cells to a series of chemotherapeutic drugs, including paclitaxel, doxorubicin, mitoxantrone, topotecan, and cisplatin. As it is shown in Fig. 6, SW620/GEDA was resistance to most of above chemotherapeutic drugs, except cisplatin. Furthermore, Ko 143, an ABCG2 specific reversal agent, could significantly reverse those drugs resistance in SW620/GEDA cells. Meanwhile, verapamil, an ABCB1 specific inhibitor, could also partially reverse SW620/GEDA cells resistance (Table 3) As it is shown in Fig. 6g, expression of ABCB1 and ABCG2 were observed in SW620/GEDA cell line.

**Table 3:** Resistance of gedatolisib in SW620/GEDA could be reversed by Ko143 and verapamil

Drug	IC_50_ ± SD
	SW620/GEDA (μΜ)
*Gedatolisib*	5.323 ± 0.728
+ Verapamil (2 μM)	4.837 ± 0.521
+ Verapamil (5 μM)	2.433 ± 0.314*
+ Ko143 (2 μM)	1.442 ± 0.238*
+ Ko143 (5 μM)	0.713 ± 0.096*
*Doxorubicin*	0.353 ± 0.042
+ Verapamil (2 μM)	0.361 ± 0.037
+ Verapamil (5 μM)	0.174 ± 0.023*
*Paclitaxel*	0.767 ± 0.062
+ Verapamil (2 μM)	0.679 ± 0.065
+ Verapamil (5 μM)	0.411 ± 0.031*
*Mitoxantrone*	0.872 ± 0.076
+ Ko143 (2 μM)	0.341 ± 0.032*
+ Ko143 (5 μM)	0.172 ± 0.011*
*Topotecan*	2.517 ± 0.329
+ Ko143 (2 μM)	1.022 ± 0.016*
+ Ko143 (5 μM)	0.239 ± 0.036*

^*^Presents when p value is < 0.05. Data was showed with mean ± SD, which is representative for at least three independent experiments

The ABCB1 and/or ABCG2 siRNA interference assays were conducted to determine the role of ABCB1 and ABCG2 on gedatolisib resistance. As it is illustrated in Additional file 1: Figure S1, transfection with ABCB1 siRNA or ABCG2 siRNA or together significantly decreased the expression level of ABCB1 and/or ABCG2. The subsequently MTT assays (Table 4) showed that, after knockdown of ABCB1 or ABCG2, the IC_50_ value of gedatolisib on SW620/GEDA significantly decreased, from 6.017 μΜ to 3.102 μΜ and 2.503 μΜ, respectively. Moreover, knockdown of both ABCB1 and ABCG2 significantly decreased the cell viability of gedatolisib in SW620/GEDA cells, even lower than that knockdown only one transporter. In addition, knockdown of ABCB1 potently lower the IC_50_ value of doxorubicin and paclitaxel in SW620/GEDA cells. Furthermore, the cell viability of SW620/GEDA cells exposed to mitoxantrone and topotecan significantly decreased after knockdown of ABCG2. However, the absence of control-siRNA did not alter any of the drugs toxicity.

**Table 4:** Resistance of gedatolisib in SW620/GEDA could be reversed by knockdown of ABCB1 and/or ABCG2

Drug	IC_50_ ± SD
	SW620/GEDA (μΜ)
*Gedatolisib*	6.017 ± 0.815
+ control-siRNA	6.124 ± 0.588
+ ABCB1-siRNA	3.102 ± 0.426*
+ ABCG2-siRNA	2.053 ± 0.301*
+ ABCB1-siRNA + ABCG2-siRNA	0.526 ± 0.078*
*Doxorubicin*	0.428 ± 0.051
+ control-siRNA	0.461 ± 0.059
+ ABCB1-siRNA	0.103 ± 0.011*
*Paclitaxel*	0.891 ± 0.093
+ control-siRNA	0.879 ± 0.077
+ ABCB1-siRNA	0.305 ± 0.042*
*Mitoxantrone*	0.936 ± 0.097
+ control-siRNA	0.925 ± 0.089
+ ABCG2-siRNA	0.136 ± 0.021*
*Topotecan*	2.905 ± 0.221
+ control-siRNA	2.798 ± 0.235
+ ABCG2-siRNA	0.217 ± 0.027*

^*^Presents when p value is < 0.05. Data was showed with mean ± SD, which is representative for at least three independent experiments

### Molecule docking of gedatolisib with modeled structure ofABCB1 and ABCG2

**Fig. 7:**
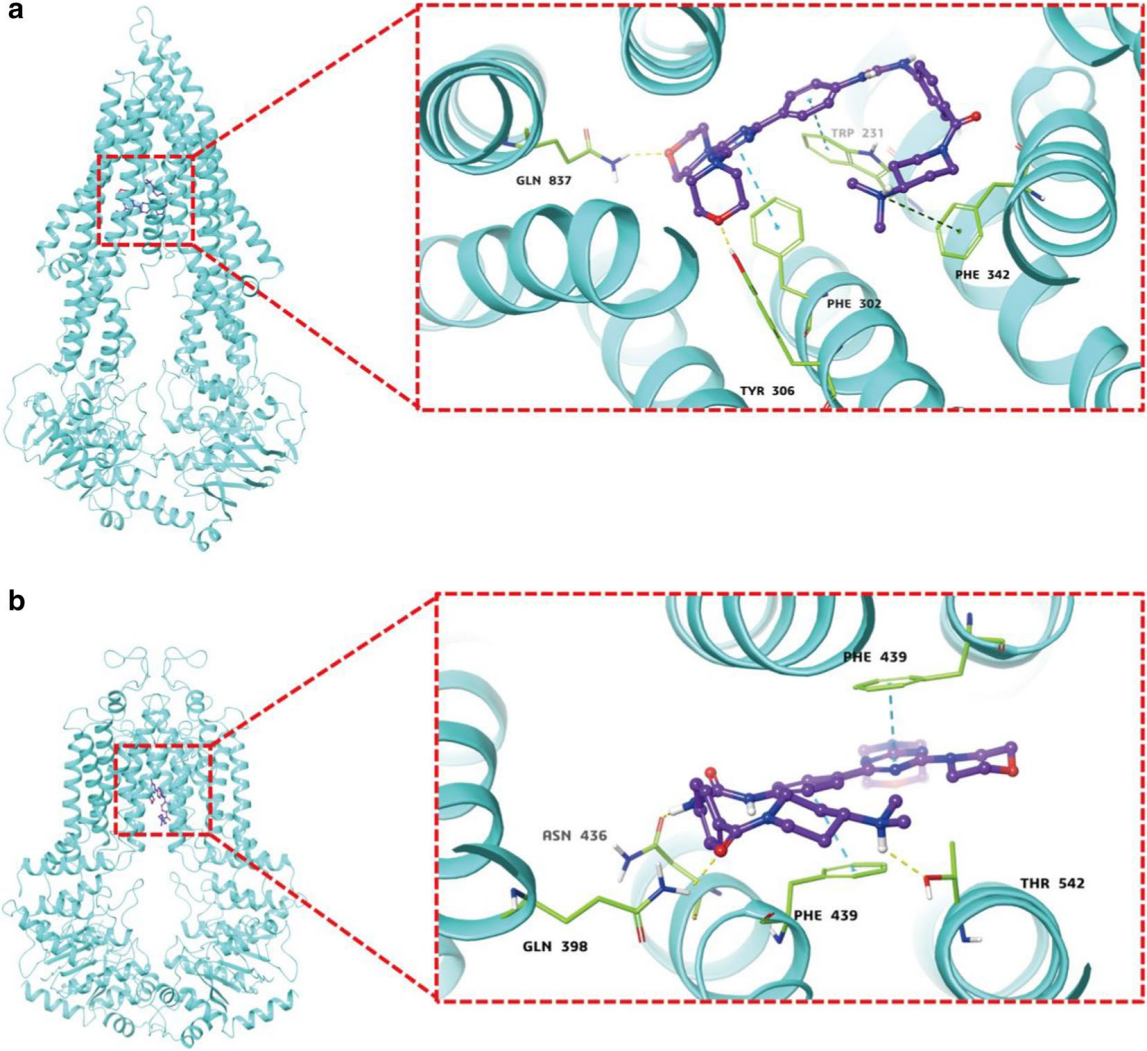
Docking of gedatolisib in the active drug binding site of ABCB1 and ABCG2. The best docked poses of gedatolisib with the residues of ABCB1 (**a**) and ABCG2 (**b**) homology model

To get further understanding of the binding site of gedatolisib with ABCB1 and ABCG2, docking analysis was performed. The active docked sites of ABCB1 and ABCG2 were shown in Fig. 7. As a result, gedatolisib could bind to active binding pocket of both ABCB1 and ABCG2, with binding score of − 9.6 kcal mol^−1^ and − 11.7 kcal mol^−1^, respectively, indicated that gedatolisib can bind to the drug-binding pockets in the trans-membrane regions of both ABCB1 and ABCG2.

## Discussion

The PI3K signaling pathways plays a central role in regulating cancer cells metabolism and survival, which is aberrantly activated in multiple types of cancer. It is reported that overactivation of PIK3CA gene mutations affecting the PI3Kα catalytic subunit, such as H1047R, P449T, E545K and R88Q, are detected in about 15–20% of CRC, which makes PI3K pathway a potential attractive target [9, 10, 25, 26]. Gedatolisib is an ATP-competitive, highly selective, and potent pan-class I isoform PI3K and mTOR inhibitor [27]. As a preclinical drug, gedatolisib has been proved to have significant anti-CRC effects [28]. For example, Gedatolisib uniting irinotecan produced a response rate of 5% and clinical benefit in 16% patients with advanced CRC (progression-free survival, 2.8 months). Preliminary evidence of clinical activity was observed with gedatolisib uniting PD-0325901 in patients with ovarian cancer (three partial responses) or endometrial cancer (one partial response) with KRAS mutations [29]. Despite the definite anticancer effects, the resistance mechanisms of gedatolisib are still remained to be declared.

Though heroic efforts to develop new anti-cancer drugs and biological therapies, as well as to investigate plenty of mechanisms of resistance to these treatment modalities, development of multidrug resistance (MDR) is still an unsolved impediment in the treatment of cancer [30]. The MDR in cancer can be induced by multiple approaches, including reduced uptake of drugs, altered cell cycle checkpoint and cell cycle arrest, altered drug targets, increased efflux of drugs by transporters, and sequestration of anticancer drugs in lysosomes as well as intracellular organelles and intercellular vesicles [31]. Among them, the predominant cause of MDR is the increased efflux of anticancer drugs by membrane-embedded ABC transporters. The ABC transporter superfamily includes a number of transporters located on the cellular plasma membrane that mediate the efflux of endogenous and exogenous substances using energy provided by ATP hydrolysis, which are grouped into seven subfamilies, designated A to G, based on their amino acid sequences [32].

Among these transporters, ABCB1 (MDR1, P-pg), ABCG2 (BCRP), and ABCC1 (MRP1) are widely investigated in past 20 years which were well-related with MDR in cancer. The overexpression of these three transporters on cancer cells would lead cancer cell resistance to a series of chemotherapeutic and target drugs, inducing drug resistance in multiple types of cancer. Moreover, in colorectal cancers, overexpression of ABCB1 and ABCG2 has been documented with poor clinical outcome [33–35]. In addition, evidence of ubiquitous expression of ABCC1 has meant it is unlikely to be a suitable target for anti-cancer therapy [36]. Hence, in present study, we focused on the effects of ABCB1 and ABCG2-mediated gedatolisib resistance in colorectal cancer cells, which may challenge its clinical administration effect.

Firstly, MTT assays were conducted to evaluate the cytotoxic effects of gedatolisib in parental CRC cell lines, and ABCB1- or ABCG2-overexpression CRC cell lines. As a result, ABCB1- and ABCG2-overexpression CRC cells showed weak responses to gedatolisib, indicating that overexpression of ABCB1 and ABCG2 might be a key factor responsible for gedatolisib resistance. Meanwhile, gedatolisib resistance were also observed when administrated in ABCB1- and ABCG2-gene-transfected HEK293 cells, while not in parental HEK293/pcDNA3.1 cell line, further verifying that overexpression of ABCB1 and ABCG2 confer resistance to gedatolisib in CRC cells. Prudently, we further evaluated whether documented ABCB1 or ABCG2 specific inhibitors could reverse gedatolisib resistance in aforementioned cell lines. Interestingly, verapamil, an ABCB1 specific inhibitor, could significantly reverse gedatolisib resistance in SW620/ADR and HEK293/ABCB1 cells. In addition, as an ABCG2 reversal agent, Ko143 significantly antagonized gedatolisib resistance in LS180/MX and HEK293/ABCG2 cells. These results confirmed that ABCB1 and ABCG2 could mediate gedatolisib resistance in CRC cell lines.

Since ABCB1 and ABCG2 are responsible for gedatolisib resistance in CRC cell lines, the mechanisms might be related with the alteration of efflux function of ABCB1 and ABCG2, videlicet, gedatolisib acts as a potential substrate of ABCB1 and ABCG2, which make it more easier to be pumped out of the ABCB1- and ABCG2-overexpression CRC cells, therefore resulted in drug resistance [37]. Subsequently, accumulation studies were conducted by tracing tritium-labeled substrate drug (paclitaxel and mitoxantrone) to indirectly indicate the accumulation level of gedatolisib in ABCB1- and ABCG2-overexpression CRC cell lines. When co-administrated with gedatolisib, the accumulation level of [^3^H]-paclitaxel in SW620/ADR cells, was significantly increased in a dose-dependent manner. However, in parental SW620 cells, the accumulation level of [^3^H]-paclitaxel was not significantly upregulated after treatment with different concentrations of gedatolisib. In addition, in LS180/MX cells, down-regulation on [^3^H]-mitoxantrone accumulation was obviously observed after co-treatment with different concentrations of gedatolisib. Nevertheless, there is no significant alteration of [^3^H]-paclitaxel or [^3^H]-mitoxantrone accumulation level in parental SW620 cells or LS180 cells after treatment with gedatolisib. These results further demonstrated that gedatolisib act as ABCB1 and ABCG2 substrates. Gedatolisib competitively increased the accumulation of tritium-labeled substrate drug, such as [^3^H]-paclitaxel and [^3^H]-mitoxantrone.

Binding domains in ABC transporters, which serve as ATPase to utilize energy from ATP hydrolysis, play a key role in drug accumulation [38]. Hence, the ATPase assays were performed to detect the impact of gedatolisib on ATPase activity of both ABCB1 and ABCG2. As we expected, gedatolisib significantly stimulated the ATPase of both ABCB1 and ABCG2, which further indicated that gedatosilib is a substrate of ABCB1 and ABCG2.

The accumulation level of substrates are also related with the expression of ABC transporters. Hence, Western blot assays were conducted to determine the influence of gedatolisib on ABCB1 and ABCG2 protein expression [39]. After treated with gedatolisib at low concentrations for 72 h, the expression of ABCB1 in SW620/ADR cells significantly increased. Meanwhile, in LS180/MX cells, ABCG2 expression level also significantly up-regulated after administrated with gedatolisib for 72 h. These results indicated that overexpression of ABCB1 and ABCG2 in CRC cells is a key factor for gedatolisib resistance. It is documented that up-regulation of ABC transporters may be involved in a series of cell signaling pathways activation. For example, the lactate receptor (HCAR1/GPR81) has been reported to be responsible for ABCB1 up-regulation in human cervical cancer cells to mediate doxorubicin resistance [40]. In colorectal cancers, upregulation of miR-199a/b has been reported to responsible for cisplatin resistance via Wnt/β-catenin-ABCG2 axis, indicated that there are a series of potential pathways which could mediate ABC transporter expression in cancers [41]. Hence, further evaluation of other potential mechanisms which mediate gedatolisib resistance in CRC cells remains to be investigated in the future. Moreover, as membrane “pumps”, the alteration of subcellular localization of ABCB1 and ABCG2 could change the efflux function. However, in our immunofluorescence assays, no significant alteration of subcellular localization of ABCB1 or ABCG2 was observed after treatment with gedatolisib, indicating that the resistance of gedatolisib was not involved in the alteration of subcellular location of ABCB1 or ABCG2.

As a big challenge in cancer therapy, ABC transporters-, especially ABCB1- and ABCG2-mediated MDR has been widely investigated in the past 3 decades, in which period a series of reversal agents have been studied and evaluated to antagonized ABCB1- or ABCG2-mediated chemotherapeutic or target drug therapy resistance [42]. Though few of those drugs work well in clinic, screening new ABC transporter inhibitors is still remained an approach to exclude MDR [38]. Some substrate drugs also act as ABC transporter reversal agent, hence, we next evaluated the reversal capacity of gedatolisib at low toxic concentrations on ABCB1- or ABCG2-mediated MDR in CRC cells [39]. However, at nearly non-toxic concentration, gedatolisib could not significantly reverse ABCB1- or ABCG2-medaited MDR in colorectal cancer cells, indicated that gedatolisib could not be a good reversal agent due to its relative high toxicity.

Establishment of resistant cell lines by increasingly administrating certain drugs could be a moderate model which can simulate a drug resistance process in vitro [43]. To get further understanding on ABCB1- and ABCG2-medaited gedatolisib resistance in CRC cells, we established a gedatolisib resistance SW620 CRC cell line, SW620/GEDA, which means in that cells, gedatolisib (3 μM) could not significantly alter the cell proliferation.

Western blot assays were conducted to evaluate the potential mechanisms contributing to gedatolisib resistance. The expression level of ABCB1 and ABCG2 in SW620/GEDA cell line were significantly increased, suggested that overexpression of ABCB1 and ABCG2 may be involved in the resistance mechanisms of gedatolisib-resistance cell line. Both of the transcriptional and post-transcriptional alteration may be involved in the upregulation of ABCB1 and/or ABCG2 [44, 45]. Furthermore, DNA methylation status has been documented to play key role in regulating the expression of the transporters. For example, hypomethylation of the ABCB1 promoter has been detected in CCRF-CEM as well as MCF-7 cell lines [46, 47]. Meanwhile, hypomethylation of the ABCG2 promoter has for example been found in ABCG2-overexpressing sublines of MCF-7, CCRF-CEM, IGROV1 and A549 cell lines [48]. These approaches provide rationale that persistently exposed by gedatolisib may induce hypomethylation of ABCB1 and/or ABCG2, which further mediate the overexpression of these transporters. As there are few reports on demethylation effect of gedatolisib, further study is still needed to verify this hypothesis. In addition, activation of Wnt/β-catenin signaling has been detected in gedatolisib-insensitive colorectal cell lines [49], based on the abundant reports that Wnt/β-catenin signaling is closely related with the expression of ABC transporters [50–56], the potential rationale of overexpression of ABCB1 and ABCG2 in gedatolisib-resistance cell line are most likely through the over-activation of Wnt/β-catenin signaling.

Our subsequently MTT results indicated that SW620/GEDA was resistant to most of ABCB1 and ABCG2 substrates, which can be reversed by ABCB1 and ABCG2 specific inhibitors, Ko143 and verapamil. However, the relative potency of ABCB1/ABCG2 inhibition by verapamil versus Ko143 may act as a key factor. Hence, we could not conclude which transporter contribute more on gedatolisib resistance. In addition, though verapamil and Ko143 were treated as inhibitors of ABCB1 and ABCG2 respectively, there are potentials that these inhibitors could exert their effects through binding to other proteins. For example, it is documented that Ko143 is not entirely specific for ABCG2 [57]. Hence, RNA-knocking down assays were conducted to exclude the false positive results made by verapamil or Ko143. After ABCB1 or ABCG2 knockdown by siRNA, the cell viability of gedatolisib in SW620/GEDA cells significantly decreased. Moreover, the much lower IC_50_ value was observed after knockdown of both ABCB1 and ABCG2 in SW620/GEDA cells, indicated that the overexpression of ABCB1 and ABCG2 are responsible for the resistance of gedatolisib. In addition, IC_50_ values of doxorubicin or paclitaxel significantly decreased after knockdown of ABCB1, while knockdown of ABCG2 potently increased the sensitivity of SW620/GEDA cells to mitoxantrone or topotecan, suggested the reality of overexpression of ABCB1 and ABCG2 in SW620/GEDA cell line.

In recent years, more ABC transporters have been identified to be related with MDR in cancer besides ABCB1, ABCG2, and ABCC1. For example, it is documented that overexpression of ABCB6 is related to resistance to 5-FU, SN-38, as well as vincristine [58]. Moreover, Zhang YK et al. have reported that, after treated with arsenic trioxide, significant overexpression of ABCB6 was found in KB cells [59]. In addition, ABCC10 was also reported to involve in the chemotherapeutic resistance in some types of cancer, including non-small cell lung cancer and ovarian cancer [60, 61]. These evidence further increase the interests on ABC transporter-mediated drug resistance in cancer. As gedatolisib is a potent pre-clinical anticancer target drug, the evaluation of ABC transporters excluded ABCB1 and ABCG2 is urgently to be further investigated in the further, which will make better understanding the mechanisms of action on gedatolisib resistance in CRC.

## Conclusions

Our study provides evidence that overexpression of ABCB1 and ABCG2 in CRC cells could be a major factor which leads to gedatolisib resistance. This means that co-administration of gedatolisib with ABCB1 or ABCG2 inhibitors may have potential benefits to avoid gedatolisib resistance and improve the efficacy of gedatolisib in colorectal cancer. Nevertheless, in vivo studied are still needed to further evaluation the safety and effectiveness that combination use of gedatolisib with ABC transporter inhibitors. In addition, other ABC transporters, such as ABCB6 and ABCC10, may also participate in the resistance of gedatolisib, which need to be investigated further in the future.

## Supplementary Information

**Additional file 1: Figure S1. **Expression level of ABCB1 and/or ABCG2 after knockdown of ABCB1 and/or ABCG2. The SW620/GEDA cell line was treated with control-siRNA, ABCB1-siRNA, and/or ABCG2-siRNA, the expression level of ABCB1 and ABCG2 were detected by Western blot assay.

## Data Availability

The datasets supporting the conclusions of this article are included within the article.

## Abbreviations

CRC: Colorectal cancer
TME: Tumor microenvironment
PI3Ks: Phosphoinositide 3-kinases
PIP2: Phosphatidylinositol 4,5-bisphosphate
PIP3: Phosphatidylinositol 3,4,5-trisphosphate
PAM: PI3K/Akt/mTOR
GSK3β: Glycogen synthesis kinase 3β
BAD: Bcl2-antagonist of cell death
FDA: The U.S. Food and Drug Administration
MDR: Multidrug resistance
ABC: ATP-binding cassette
DMEM: Dulbecco’s modified eagle’s medium
MTT: 3-4,5-Dimethylthiazol-yl-2,5-diphenyltetrazolium bromide
PI: Propidium iodide
