# Supplementary figures and images for: ABCB1 and ABCG2 restricts the efficacy of gedatolisib (PF-05212384), a PI3K inhibitor in colorectal cancer cells
Source: Cancer Cell Int. 2021 Feb 16;21:108. doi: 10.1186/s12935-021-01800-7 (PMC7885361; doi:10.1186/s12935-021-01800-7)

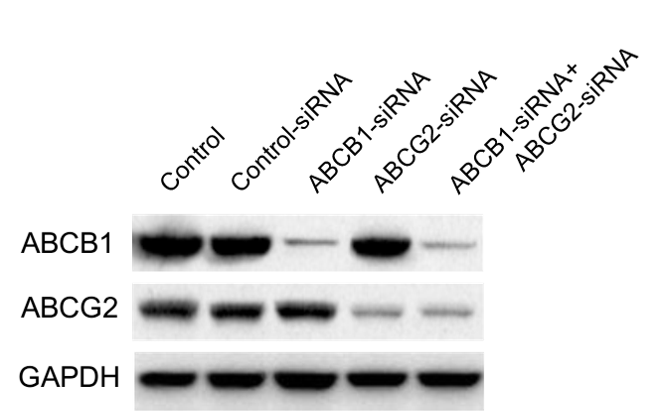

Supplement: Supplementary file 1 — Additional file 1: Figure S1. Expression level of ABCB1 and/or ABCG2 after knockdown of ABCB1 and/or ABCG2. The SW620/GEDA cell line was treated with control-siRNA, ABCB1-siRNA, and/or ABCG2-siRNA, the expression level of ABCB1 and ABCG2 were detected by Western blot assay. [file 12935_2021_1800_MOESM1_ESM.png]
